# Supplementary figures and images for: Genetic variants associated with sepsis
Source: PLoS One. 2022 Mar 11;17(3):e0265052. doi: 10.1371/journal.pone.0265052 (PMC8916629; doi:10.1371/journal.pone.0265052)

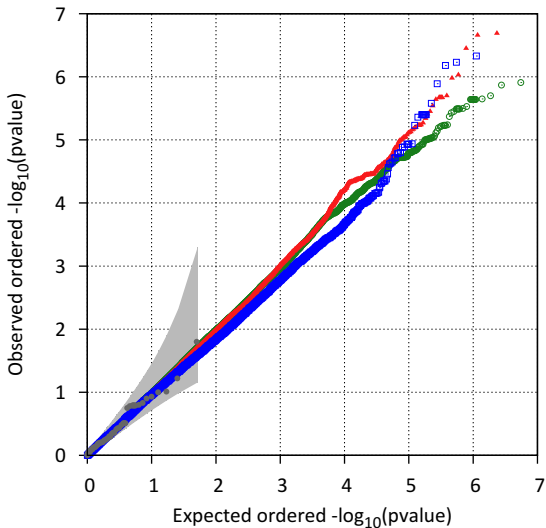

MAF [.05,.5] (5595196)  
MAF [.01,.05] (2368244)  
MAF [.005,.01] (1123702)  
MAF [.001,.005] (51)

Supplement: S1 Fig — MAF—minor allele frequency. (PDF) [file pone.0265052.s001.pdf]

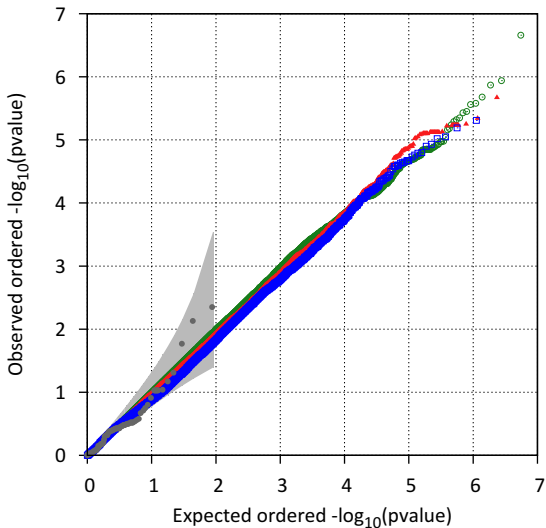

MAF [.05,.5] (5590060)  
MAF [.01,.05] (2367383)  
MAF [.005,.01] (1124847)  
MAF [.001,.005] (89)

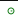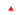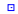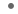

Supplement: S2 Fig — MAF—minor allele frequency. (PDF) [file pone.0265052.s002.pdf]
