# Supplementary material for: Genetic variants associated with sepsis
Source: PLoS One. 2022 Mar 11;17(3):e0265052. doi: 10.1371/journal.pone.0265052 (PMC8916629; doi:10.1371/journal.pone.0265052)
Supplement: S1 Table — (DOCX) [file pone.0265052.s003.docx]

| 001-005 | 491.21 | 599 |
| --- | --- | --- |
| 008-018 | 494 | 601 |
| 020-027 | 510 | 604 |
| 030-041 | 513 | 614-616 |
| 090-098 | 522.5 | 646.6 |
| 100-104 | 522.7 | 658.4 |
| 110-112 | 526.4 | 670 |
| 114-118 | 527.3 | 675.1 |
| 120.99 | 540-542 | 681-683 |
| 320 | 562.01 | 685 |
| 322 | 562.03 | 686 |
| 324-325 | 562.11 | 711 |
| 360 | 562.13 | 728.86 |
| 376 | 566-567 | 730 |
| 380.14 | 569.5 | 785.52 |
| 383 | 569.61 | 790.7 |
| 420-421 | 569.83 | 958.3 |
| 451 | 572 | 995.90-995.92 |
| 461-465 | 572.1 | 996.6 |
| 475 | 575 | 998.5 |
| 481-482 | 590 | 999.3 |
| 485-486 | 597 |  |

S1 Table 1. ICD9 codes suggestive of infection that were used to remove possible control patients.
