# Supplementary material for: Genetic variants associated with sepsis
Source: PLoS One. 2022 Mar 11;17(3):e0265052. doi: 10.1371/journal.pone.0265052 (PMC8916629; doi:10.1371/journal.pone.0265052)
Supplement: S2 Table — C-statistic = 0.684 (95%confidence interval = 0.672–0.696) BOLD identify genetic variants associated with both Sepsis-2 and Sepsis-3 sepsis. Factor shows the odds ratios for each of the other components of the analysis. PC–principal component. (DOCX) [file pone.0265052.s004.docx]

| Factor | OR | 95% CI | p-value |  |  |
| --- | --- | --- | --- | --- | --- |
| Intercept | 0.08 | (0.03, 0.20 ) | <0.0001 |  |  |
| Inferred Female | 0.83 | (0.75, 0.90 ) | <0.0001 |  |  |
| PC1 | 0 | (0, 1.16) | 0.0547 |  |  |
| PC2 | 58.7 | (0.63, 5429) | 0.0779 |  |  |
| PC3 | 0 | (0, 0.01) | 0.0031 |  |  |
| PC4 | 1549 | (0, 9x10^14^) | 0.5952 |  |  |
| Gene | OR | 95% CI | p-value | Gene name | Variant |
| ***IL12RB1*** | 2.63 | (1.57, 4.42) | 0.0003 | Interleukin 12 receptor subunit beta | 19:18184213:T:A |
| PPARG | 2.37 | (1.51, 3.72) | 0.0002 | Peroxisome proliferator-activated receptor gamma | 3:12389471:G:T |
| GAS6 | 2.21 | (1.21, 4.04) | 0.0098 | Growth arrest specific 6 | 13:114543624:C:G |
| MAPK14 | 2.03 | (1.36, 3.02) | 0.0005 | Mitogen-activated protein kinase 14 | 6:36044669:G:A |
| ATF3 | 2.00 | (1.34, 2.98) | 0.0007 | Activating transcription factor 3 | 1:212753582:A:G |
| ***NFKB1*** | 1.98 | (1.29, 3.04) | 0.0018 | Nuclear factor kappa B subunit 1 | 4:103430587:C:T |
| ***SDC1*** | 1.89 | (1.28, 2.80) | 0.0015 | Syndecan 1 | 2:20421871:C:G |
| ***NTN1*** | 1.87 | (1.25, 2.80) | 0.0024 | Netrin 1 | 17:9138239:C:T |
| ***PPARA*** | 1.86 | (1.27, 2.72) | 0.0014 | Peroxisome proliferator-activated receptor alpha | 22:46579337:A:T |
| ***NTN1*** | 1.85 | (1.16, 2.97) | 0.0101 | Netrin 1 | 17:9088553:G:A |
| TNFRSF14 | 1.84 | (1.20, 2.83) | 0.0054 | Tumor necrosis factor receptor associated factor 14 | 1:2492641:G:T |
| ***PCSK9*** | 1.84 | (1.18, 2.86) | 0.0070 | Proprotein convertase subtilisin/kexin type 9 | 1:55514952:G:A |
| ***PBX3*** | 1.83 | (1.21, 2.78) | 0.0045 | PBX homeobox 3 | 9:128597167:G:A |
| MARCO | 1.79 | (1.14, 2.80) | 0.0116 | Macrophage receptor with collagenous structure | 2:119721143:T:C |
| ***MLKL*** | 1.75 | (1.33, 2.31) | <0.0001 | Mixed lineage kinase domain like pseudokinase | 16:74731984:T:A |
| ***FAS*** | 1.71 | (1.10, 2.66) | 0.0177 | Fas cell surface death receptor | 10:90750339:G:A |
| ALB | 1.71 | (1.19, 2.45) | 0.0035 | Albumin | 4:74273913:G:A |
| PAPPA | 1.70 | (1.12, 2.60) | 0.0136 | Pappalysin 1 | 9:118947383:T:C |
| ***TIMP2*** | 1.69 | (1.08, 2.63) | 0.0214 | Tissue inhibitor of metallopeptidases 2 | 17:76885822:A:G |
| TFPI | 1.68 | (1.28, 2.21) | 0.0002 | Tissue factor pathway inhibitor | 2:188369452:T:G |
| ***F2R*** | 1.67 | (1.20, 2.32) | 0.0021 | Coagulation Factor II thrombin receptor | 5:76015847:A:G |
| MARCO | 1.66 | (1.23, 2.23) | 0.0010 | Macrophage receptor with collagenous structure | 2:119736539:G:A |
| VCAM1 | 1.63 | (1.21, 2.20) | 0.0012 | Vascular cell adhesion molecule 1 | 1:101188440:G:C |
| NAMPT | 1.60 | (1.20, 2.14) | 0.0014 | Nicotinamide phosphoribosyltransferase | 7:105908425:G:C |
| EDN1 | 1.57 | (1.17, 2.12) | 0.0028 | Endothelin 1 | 6:12267550:T:C |
| MYLK | 1.57 | (1.07, 2.30) | 0.0208 | Myosine light chain kiinase | 3:123429504:T:A |
| AGRN | 1.56 | (1.15, 2.12) | 0.0046 | Agrin | 1:958204:C:A |
| ***ADGRE2*** | 1.56 | (1.21, 2.02) | 0.0007 | Adhesion G protein-coupled receptor E2 | 19:14871817:C:T |
| GLP1R | 1.56 | (1.13, 2.15) | 0.0075 | Glucagon like peptide 1 | 6:39023903:C:T |
| ***CX3CR1*** | 1.55 | (1.15, 2.08) | 0.0041 | C-X-C motif chemokine receptor 1 | 3:39306134:C:T |
| TF | 1.54 | (1.11, 2.14) | 0.0106 | Transferrin | 3:133456635:T:A |
| BCL2L11 | 1.50 | (1.14, 1.97) | 0.0037 | BCT-2-like protein 1 | 2:111900451:A:G |
| ***IGF1*** | 1.48 | (1.00, 2.21) | 0.0529 | Insulin Like Growth Factor 1 | 12:102797525:C:A |
| ***SCN1A*** | 1.48 | (1.20, 1.82) | 0.0002 | Sodium channelt, type 1, alpha subunit | 2:166922213:T:C |
| ATF3 | 1.42 | (1.09, 1.86) | 0.0104 | Activating transcription factor 3 | 1:212762164:C:T |
| PLAUR | 1.41 | (1.09, 1.84) | 0.0102 | Urokinase plaminogen activator surface receptor | 19:44152286:T:A |
| TGFB1 | 1.40 | (1.17, 1.68) | 0.0003 | Transforming growth factor beta 1 | 19:41847860:G:A |
| ***TGM2*** | 1.39 | (1.12, 1.72) | 0.0025 | Transglutiminase 2 | 20:36783114:G:A |
| MAPK14 | 1.39 | (1.12, 1.71) | 0.0023 | Mitogen-activated protein kinase 14 | 6:35999843:G:A |
| VCAM1 | 1.38 | (1.09, 1.76) | 0.0080 | Vascular cell adhesion molecule 1 | 1:101197384:A:G |
| ***PDE4B*** | 1.38 | (0.98, 1.95) | 0.0658 | Phosphodiesterase 4B | 1:66837281:C:T |
| PGF | 1.37 | (1.11, 1.70) | 0.0030 | Placental growth factor | 14:75411057:G:A |
| ***IL32*** | 1.36 | (1.07, 1.73) | 0.0129 | Interleukin 32 | 16:3116295:A:G |
| GSN | 1.36 | (1.13, 1.64) | 0.0014 | Gelsolin | 9:123964830:C:T |
| AMPD1 | 1.35 | (1.08, 1.68) | 0.0090 | adenosine monophosphate deaminase 1 | 1:115217148:A:G |
| ***TGM2*** | 1.33 | (1.09, 1.63) | 0.0046 | Transglutiminase 2 | 20:36774436:G:A |
| ***FLT1*** | 1.32 | (1.10, 1.58) | 0.0030 | vascular endothelial growth factor receptor 1 | 13:29054670:T:C |
| CD86 | 1.31 | (1.09, 1.58) | 0.0043 | T-lymphocyte activation antigen | 3:121804804:C:T |
| SPOCK1 | 1.31 | (1.06, 1.61) | 0.0132 | Testican-1 | 5:136566815:C:T |
| HSPA4 | 1.30 | (1.07, 1.57) | 0.0083 | Heat shock protein family A member 4 | 5:132407956:G:A |
| ADAMTS13 | 1.25 | (1.04, 1.49) | 0.0168 | ADAM Metallopeptidase with thrombospondin Type 1 Motif 13 | 9:136303993:T:C |
| ***PDE4B*** | 1.25 | (0.99, 1.56) | 0.0579 | Phosphodiesterase 4B | 1:66605189:G:T |
| IGF1 | 1.18 | (1.05, 1.32) | 0.0052 | Insulin Like Growth Factor 1 | 12:102789852:C:G |
| ***PDE4B*** | 1.17 | (0.98, 1.39) | 0.0858 | Phosphodiesterase 4B | 1:66339323:T:C |
| ***VWF*** | 1.16 | (1.06, 1.28) | 0.0018 | Von Willebrand Factor | 12:6190529:C:T |
| AGER | 1.16 | (1.05, 1.29) | 0.0046 | Advanced Glycosylation End-product specific receptor | 6:32151994:A:T |
| SERPINA1 | 1.16 | (1.06, 1.27) | 0.0009 | Serpin family A member 1 | 14:94849882:T:C |
| ***PYGM*** | 1.15 | (1.04, 1.27) | 0.0057 | Glycogen phospholorase (myophosphorlase) | 11:64520255:C:G |
| ITGA4 | 1.13 | (1.04, 1.24) | 0.0060 | Integrin subunit alpha 4 | 2:182355984:G:A |
| ***PLG*** | 1.13 | (1.05, 1.22) | 0.0019 | Plasminogen | 6:161163074:A:T |
| ***CXCL12*** | 1.11 | (1.02, 1.20) | 0.0121 | C-X-C motif chemokine 12 / stromal cell-derived factor 1 | 10:44859439:A:G |
| TFPI | 1.11 | (1.03, 1.19) | 0.0032 | Tissue factor pathway inhibitor | 2:188401301:A:G |
| PPARG | 1.10 | (1.03, 1.18) | 0.0049 | Peroxisome proliferator-activated receptor gamma | 3:12508598:A:G |
| PLAT | 1.09 | (1.02, 1.18) | 0.0156 | Tissue type plasminogen activator | 8:42047302:G:A |
| ***PRL*** | 1.09 | (1.01, 1.16) | 0.0171 | Prolactin | 6:22289461:T:C |
| ***IL12RB1*** | 0.94 | (0.87, 1.02) | 0.1495 | Interleukin 12 receptor subunit beta | 19:18187562:G:A |
| ***ANGPT2*** | 0.92 | (0.86, 0.98) | 0.0115 | Angiopoietin 2 | 8:6361229:A:C |
| ***PDE4B*** | 0.91 | (0.84, 0.98) | 0.0173 | Phosphodiesterase 4B | 1:66736066:C:T |
| ***IL12RB1*** | 0.90 | (0.83, 0.98) | 0.0191 | Interleukin 12 receptor subunit beta | 19:18193613:A:G |
| ***TEK*** | 0.90 | (0.84, 0.96) | 0.0021 | Angiopoietin 1 receptor | 9:27168704:T:C |
| SELP | 0.90 | (0.83, 0.98) | 0.0143 | Selectin P | 1:169580885:C:T |
| TRAF6 | 0.89 | (0.82, 0.98) | 0.0122 | Tumor necrosis factor receptor associated factor 6 | 11:36530644:C:G |
| ***GZMK*** | 0.89 | (0.83, 0.96) | 0.0026 | Granzyme K | 5:54322476:C:G |
| SPOCK1 | 0.89 | (0.82, 0.97) | 0.0057 | Testican-1 | 5:136388933:C:T |
| FLT1 | 0.89 | (0.82, 0.96) | 0.0046 | vascular endothelial growth factor receptor 1 | 13:28883337:A:G |
| TRB | 0.88 | (0.81, 0.96) | 0.0030 | T cell receptor beta locus | 7:142272563:C:T |
| MDM2 | 0.88 | (0.82, 0.95) | 0.0011 | Mouse double minute 2 (E3 ubiquitin-protein ligase) | 12:69218038:T:G |
| GHR | 0.87 | (0.79, 0.96) | 0.0043 | Growth hormone receptor | 5:42592285:C:T |
| PLG | 0.86 | (0.72, 1.04) | 0.1135 | Plasminogen | 6:161145234:T:C |
| CD22 | 0.84 | (0.74, 0.96) | 0.0111 | Cluster of differentiation 22 | 19:35831435:A:G |
| CHGA | 0.82 | (0.71, 0.94) | 0.0043 | Chromagranin A | 14:93394901:T:C |
| ***PCSK9*** | 0.80 | (0.67, 0.95) | 0.0112 | Proprotein convertase subtilisin/kexin type 9 | 1:55521352:C:T |
| NR1I2 | 0.76 | (0.64, 0.90) | 0.0019 | Nuclear receptor subfamily 1 group 1 member 2 | 3:119505675:C:T |
| TNFSF4 | 0.75 | (0.63, 0.90) | 0.0019 | Tumor necrosis factor superfamily member 4 | 1:173299064:G:A |
| GHR | 0.74 | (0.61, 0.90) | 0.0025 | Growth hormone receptor | 5:42573791:G:A |
| ITGAX | 0.74 | (0.59, 0.93) | 0.0096 | Integrin subunit alpha X | 16:31375234:C:T |
| ***PROCR*** | 0.72 | (0.55, 0.95) | 0.0183 | Protein C receptor | 20:33776612:G:T |
| ***TIMP2*** | 0.71 | (0.56, 0.91) | 0.0070 | Tissue inhibitor of metallopeptidases 2 | 17:76865707:A:G |
| HSPA4 | 0.71 | (0.54, 0.93) | 0.0136 | Heat shock protein family A member 4 | 5:132399205:C:T |
| PAPPA | 0.70 | (0.57, 0.85) | 0.0004 | Pappalysin 1 | 9:118970563:A:G |
| FLT1 | 0.67 | (0.49, 0.92) | 0.0137 | Vascular endothelial growth factor receptor 1 | 13:29039845:T:C |
| CAV1 | 0.65 | (0.50, 0.85) | 0.0020 | Caveolin 1 | 7:116190817:A:G |
| PDE7A | 0.65 | (0.48, 0.87) | 0.0034 | Phosphodiesterase 7 | 8:66668611:A:C |
| LY96 | 0.54 | (0.38, 0.76) | 0.0005 | Lymphocyte antigen 96 | 8:74910274:G:A |
| LOC101929258 | 0.52 | (0.33, 0.84) | 0.0073 | Unnamed | 8:23576564:G:T |
| MYLK | 0.52 | (0.32, 0.84) | 0.0081 | Myosin light chain kinase | 3:123356070:C:T |
| SST | 0.48 | (0.29, 0.82) | 0.0065 | Somatostatin | 3:187388048:G:A |
| CD276 | 0.48 | (0.30, 0.75) | 0.0015 | Cluster of differentiation 276 | 15:73981849:A:C |
| LTF | 0.45 | (0.26, 0.79) | 0.0052 | Lactoferrin | 3:46525496:C:A |
| FLT1 | 0.41 | (0.20, 0.85) | 0.0159 | vascular endothelial growth factor receptor 1 | 13:28885760:G:A |
| ***TJP1*** | 0.29 | (0.14, 0.56) | 0.0003 | Tight junction protein 1 | 15:30181207:C:T |

S2 Table. Variants associated with Sepsis-2. C-statistic = 0.684 (95%confidence interval = 0.672 – 0.696) ***BOLD*** identify genetic variants associated with both Sepsis-2 and Sepsis-3 sepsis. Factor shows the odds ratios for each of the other components of the analysis. PC – principal component.
