# Supplementary material for: Genetic variants associated with sepsis
Source: PLoS One. 2022 Mar 11;17(3):e0265052. doi: 10.1371/journal.pone.0265052 (PMC8916629; doi:10.1371/journal.pone.0265052)
Supplement: S3 Table — C-statistic = 0.693 (95%confidence interval = 0.678–0.707) BOLD identify genetic variants associated with both Sepsis-2 and Sepsis-3 sepsis. Factor shows the odds ratios for each of the other components of the analysis. PC–principal component. (DOCX) [file pone.0265052.s005.docx]

| Factor | OR | 95% CI | p-value |  |  | |  |
| --- | --- | --- | --- | --- | --- | --- | --- |
| Intercept | 0.05 | (0.03, 0.12) | <0.0001 |  |  | |  |
| Inferred Female | 0.656 | (0.585, 0.735) | <0.0001 |  |  | |  |
| PC1 | .0056 | (10^-6^, 43.67) | 0.2565 |  |  | |  |
| PC2 | .00033 | (10^-6^, 0.093) | 0.0054 |  |  | |  |
| PC3 | 0 | (0, 0.0023 | 0.0023 |  |  | |  |
| PC4 | 45791 | (0, 9.3x10^19^) | 0.5861 |  |  | |  |
| Gene | OR | 95% CI | p-value | Gene name | | Variant | |
| ***PPARA*** | 2.30 | (1.5, 3.52) | 0.0001 | Peroxisome proliferator-activated receptor alpha | | 22:46579337:A:T | |
| MLKL | 2.20 | (1.38, 3.49) | 0.0009 | Mixed lineage kinase domain like pseudokinase | | 16:74721868:T:C | |
| ANGPT2 | 2.20 | (1.32, 3.64) | 0.0023 | Angiopoietin 2 | | 8:6395819:C:T | |
| ***TIMP2*** | 2.06 | (1.24, 3.41) | 0.0052 | Tissue inhibitor of metallopeptidases 2 | | 17:76885822:A:G | |
| ***NFKB1*** | 2.04 | (1.23, 3.36) | 0.0056 | Nuclear factor kappa B subunit 1 | | 4:103430587:C:T | |
| ***SDC1*** | 2.03 | (1.26, 3.28) | 0.0037 | Syndecan 1 | | 2:20421871:C:G | |
| ANGPT2 | 1.96 | (1.27, 3.04) | 0.0025 | Angiopoietin 2 | | 8:6398203:G:A | |
| FAS | 1.94 | (1.21, 3.12) | 0.0058 | Fas cell surface death receptor | | 10:90754810:A:G | |
| ***F2R*** | 1.94 | (1.32, 2.83) | 0.0006 | Coagulation Factor II thrombin receptor | | 5:76015847:A:G | |
| ***PCSK9*** | 1.93 | (1.15, 3.25) | 0.0134 | Proprotein convertase subtilisin/kexin type 9 | | 1:55514952:G:A | |
| ***NTN1*** | 1.92 | (1.18, 3.14) | 0.0090 | Netrin 1 | | 17:9138239:C:T | |
| LTA | 1.86 | (1.21, 2.85) | 0.0048 | Lymphotoxin alpha | | 6:31528501:T:C | |
| CD4 | 1.83 | (1.25, 2.70) | 0.0021 | CD4 protein | | 12:6907971:C:G | |
| IRGM | 1.82 | (1.21, 2.72) | 0.0037 | Immunity-related GTPase family M protein | | 5:150253800:G:C | |
| PBX3 | 1.78 | (1.26, 2.50) | 0.0009 | PBX homeobox 3 | | 9:128535780:C:T | |
| ***MLKL*** | 1.75 | (1.25, 2.44) | 0.0011 | Mixed lineage kinase domain like pseudokinase | | 16:74731984:T:A | |
| TRB | 1.69 | (1.15, 2.48) | 0.0073 | T cell receptor beta locus | | 7:142496944:T:A | |
| HPSE | 1.69 | (1.11, 2.56) | 0.0145 | Heparanase | | 4:84235998:A:G | |
| SPOCK1 | 1.64 | (1.14, 2.36) | 0.0083 | Testican-1 | | 5:136517628:C:T | |
| ***IL32*** | 1.56 | (1.17, 2.06) | 0.0021 | Interleukin 32 | | 16:3116295:A:G | |
| ***SPOCK1*** | 1.54 | (1.2, 1.97) | 0.0006 | Testican-1 | | 5:136566815:C:T | |
| MMP1 | 1.50 | (1.14, 1.96) | 0.0036 | Matrix metallopeptidase 1 | | 11:102661425:T:C | |
| NINJ1 | 1.48 | (1.11, 1.96) | 0.0079 | Ninjurin 1 | | 9:95885855:C:T | |
| ***SCN1A*** | 1.46 | (1.13, 1.88) | 0.0040 | Sodium channel, type 1, alpha subunit | | 2:166922213:T:C | |
| IL12RB1 | 1.45 | (1.19, 1.76) | 0.0002 | Interleukin 12 receptor subunit beta | | 19:18198923:C:T | |
| ***TGM2*** | 1.42 | (1.1, 1.84) | 0.0074 | Transglutiminase 2 | | 20:36783114:G:A | |
| ***TGM2*** | 1.40 | (1.1, 1.77) | 0.0066 | Transglutiminase 2 | | 20:36774436:G:A | |
| LTF | 1.39 | (1.02, 1.88) | 0.0367 | Lactoferrin | | 3:46483351:G:A | |
| ICAM1 | 1.39 | (1.15, 1.68) | 0.0007 | Intercellular adhesion molecule 1 | | 19:10391666:T:C | |
| ***PDE4B*** | 1.38 | (1.14, 1.67) | 0.0008 | Phosphodiesterase 4B | | 1:66339323:T:C | |
| PDE4A | 1.32 | (1.1, 1.59) | 0.0025 | Phosphodiesterase 4A | | 19:10565651:G:C | |
| ***FLT1*** | 1.32 | (1.06, 1.63) | 0.0134 | vascular endothelial growth factor receptor 1 | | 13:29054670:T:C | |
| CD4 | 1.31 | (1.05, 1.62) | 0.0152 | CD4 protein | | 12:6913546:A:G | |
| ZBTB7C | 1.27 | (1.08, 1.48) | 0.0041 | Zinc finger and BTB domain containing 7C | | 18:45780746:A:G | |
| SERPINA12 | 1.25 | (1.09, 1.44) | 0.0020 | Serpin family A member 12 | | 14:94967641:C:T | |
| NTN1 | 1.25 | (1.06, 1.46) | 0.0065 | Netrin 1 | | 17:9122038:C:T | |
| LTF | 1.23 | (1.06, 1.43) | 0.0080 | Lactoferrin | | 3:46522279:C:T | |
| ***IGF1*** | 1.23 | (1.08, 1.39) | 0.0015 | Insulin Like Growth Factor 1 | | 12:102789852:C:G | |
| ***PYGM*** | 1.21 | (1.08, 1.36) | 0.0016 | Glycogen phospholorase (myophosphorlase) | | 11:64520255:C:G | |
| HMGB1 | 1.21 | (1.05, 1.39) | 0.0092 | High mobility group box 1 | | 13:31113379:T:G | |
| BCKDK | 1.20 | (1.06, 1.35) | 0.0035 | Branched chain keto acid dehydrogenase kinase | | 16:31126688:C:A | |
| ***CXCL12*** | 1.20 | (1.09, 1.31) | 0.0003 | C-X-C motif chemokine 12 | | 10:44859439:A:G | |
| TFF1 | 1.18 | (1.08, 1.29) | 0.0004 | Trefoil 1 | | 21:43786512:C:T | |
| DPP4 | 1.18 | (1.03, 1.34) | 0.0201 | Dipeptidyl pepditase-4 (CD26) | | 2:162866992:C:T | |
| PDE4B | 1.16 | (1.03, 1.29) | 0.0106 | Phosphodiesterase 4B | | 1:66625219:T:C | |
| ADGRE2 | 1.14 | (1.04, 1.25) | 0.0061 | Adhesion G protein-coupled receptor E2 | | 19:14888054:T:C | |
| VWF | 1.14 | (1.03, 1.24) | 0.0082 | Von Willebrand Factor | | 12:6172348:C:A | |
| ***PLG*** | 1.13 | (1.03, 1.24) | 0.0104 | Plasminogen | | 6:161163074:A:T | |
| PLG | 1.13 | (0.99, 1.28) | 0.0667 | Plasminogen | | 6:161149356:C:T | |
| ZBTB7C | 1.12 | (1.03, 1.21) | 0.0108 | Zinc finger and BTB domain containing 7C | | 18:45606093:T:C | |
| BPI | 1.11 | (1.02, 1.21) | 0.0114 | Bactericidal permeability increasing protein | | 20:36937881:T:A | |
| ***PRL*** | 1.10 | (1.02, 1.20) | 0.0187 | Prolactin | | 6:22289461:T:C | |
| ***TEK*** | 0.91 | (0.83, 0.98) | 0.0180 | Angiopoietin 1 receptor | | 9:27168704:T:C | |
| CX3CR1 | 0.90 | (0.83, 0.98) | 0.0189 | C-X-C motif chemokine receptor 1 | | 3:39311666:C:T | |
| ***GZMK*** | 0.90 | (0.82, 0.98) | 0.0187 | Granzyme K | | 5:54322476:C:G | |
| DPP4 | 0.89 | (0.81, 0.97) | 0.0146 | Dipeptidyl pepditase-4 (CD26) | | 2:162913166:T:C | |
| ***MDM2*** | 0.89 | (0.81, 0.97) | 0.0128 | Mouse double minute 2 (E3 ubiquitin-protein ligase) | | 12:69218038:T:G | |
| SUFU | 0.89 | (0.81, 0.97) | 0.0098 | Suppressor of fused homolog | | 10:104318966:A:G | |
| CX3CR1 | 0.88 | (0.78, 0.99) | 0.0373 | C-X-C motif chemokine receptor 1 | | 3:39322665:A:G | |
| F5 | 0.86 | (0.78, 0.93) | 0.0009 | Coagulation factor V | | 1:169522631:T:A | |
| CD274 | 0.85 | (0.77, 0.94) | 0.0034 | Programmed death-ligand 1 | | 9:5465732:G:A | |
| TRB | 0.78 | (0.65, 0.94) | 0.0127 | T cell receptor beta locus | | 7:142496622:T:C | |
| IL2RA | 0.75 | (0.64, 0.88) | 0.0007 | Interleukin 2 receptor subunit alpha | | 10:6053866:C:T | |
| ZBTB7C | 0.71 | (0.54, 0.92) | 0.0114 | Zinc finger and BTB domain containing 7C | | 18:45565101:C:T | |
| ***PROCR*** | 0.59 | (0.43, 0.80) | 0.0010 | Protein C receptor | | 20:33776612:G:T | |
| TLR5 | 0.46 | (0.28, 0.76) | 0.0023 | Toll-like receptor 5 | | 1:223301612:C:T | |
| ***TJP1*** | 0.34 | (0.16, 0.74) | 0.0071 | Tight junction protein 1 | | 15:30181207:C:T | |
| VWF | 0.29 | (0.13, 0.65) | 0.0030 | Von Willebrand Factor | | 12:6170467:C:T | |
| CHRNA7 | 0.12 | (0.02, 0.56) | 0.0076 | cholinergic receptor nicotinic alpha 7 subunit | | 15:32413847:G:A | |

S3 Table. Variants associated with Sepsis-3. C-statistic = 0.693 (95%confidence interval = 0.678 – 0.707) ***BOLD*** identify genetic variants associated with both Sepsis-2 and Sepsis-3 sepsis. Factor shows the odds ratios for each of the other components of the analysis. PC – principal component.
