# Supplementary material for: Genetic variants associated with sepsis
Source: PLoS One. 2022 Mar 11;17(3):e0265052. doi: 10.1371/journal.pone.0265052 (PMC8916629; doi:10.1371/journal.pone.0265052)
Supplement: S4 Table — C-statistic = 0.721 (95% confidence interval = 0.671–0.768). PC–principal component. (DOCX) [file pone.0265052.s006.docx]

| Factor | OR | 95% CI | p-value |  |  |
| --- | --- | --- | --- | --- | --- |
| Intercept | 0.01 | (0.00, 0.10) | <0.001 |  |  |
| Inferred Female | 0.71 | (0.48, 1.05) | 0.083 |  |  |
| PC1 | 0.00 | (0, 8.50x10^18^) | 0.564 |  |  |
| PC2 | 78207 | (0, 6.85x10^19^) | 0.521 |  |  |
| PC3 | 0.00 | (0, 1.45x10^9^) | 0.218 |  |  |
| PC4 | 1.26x10^26^ | (0, 7.52x10^86^) | 0.555 |  |  |
| Gene | OR | 95% CI | p-value | Gene name | Variant |
| PDE4B | 2.06 | (1.33, 3.19) | 0.001 | Phosphodiesterase 4B | 1:66277843:G:A |
| PDE4B | 6.08 | (1.92, 19.3) | 0.002 | Phosphodiesterase 4B | 1:66427724:C:T |
| CFH | 4.53 | (1.87, 10.97) | 0.001 | complement factor H | 1:196634467:T:A |
| HLA-DRA | 0.63 | (0.47, 0.86) | 0.003 | Major Histocompatibility Complex, Class II, **DR** Alpha | 6:32408735:A:C |
| NAMPT | 10.61 | (2.47, 45.64) | 0.002 | Nicotinamide Phosphoribosyltransferase | 7:105901555:C:T |
| ITGB1 | 2.60 | (1.34, 5.03) | 0.005 | Integrin Subunit Beta 1 | 10:33190652:A:G |
| STIM1 | 1.94 | (1.27, 2.96) | 0.002 | Stromal Interaction Molecule 1 | 11:4093480:A:G |
| CHRNA7 | 2.10 | (1.25, 3.53) | 0.005 | Cholinergic Receptor Nicotinic Alpha 7 Subunit | 15:32442808:A:G |
| MMP2 | 1.47 | (1.12, 1.94) | 0.006 | Matrix Metallopeptidase 2 | 16:55534078:G:A |
| PRTN3 | 3.91 | (1.34, 11.44) | 0.013 | Proteinase 3 | 19:845535:C:G |
| TGM2 | 0.66 | (0.45, 0.97) | 0.032 | Transglutaminase 2 | 20:36791204:G:T |

S4 Table. Variants associated with mortality. C-statistic = 0.721 (95% confidence interval = 0.671 – 0.768). PC – principal component.
