# Supplementary material for: Genetic variants associated with sepsis
Source: PLoS One. 2022 Mar 11;17(3):e0265052. doi: 10.1371/journal.pone.0265052 (PMC8916629; doi:10.1371/journal.pone.0265052)
Supplement: Attachment — Submitted filename: Response.docx [file pone.0265052.s007.docx]

Journal Requirements:

1. When submitting your revision, we need you to address these additional requirements.

Please ensure that your manuscript meets PLOS ONE's style requirements, including those for file naming. The PLOS ONE style templates can be found at 
<https://journals.plos.org/plosone/s/file?id=wjVg/PLOSOne_formatting_sample_main_body.pdf> and 
<https://journals.plos.org/plosone/s/file?id=ba62/PLOSOne_formatting_sample_title_authors_affiliations.pdf>

Response: We have made the style changes.

2.Thank you for stating the following in the Competing Interests section: 
(Nicholas Douville was supported by a Foundation for Anesthesia Education and Research (FAER) Mentored Research Training Grant
Michael Maile was supported by the American Diabetes Association.
Milo Engoren has received consulting fee from Aerogen and Masimo.
The other authors have nothing to declare.)

Please confirm that this does not alter your adherence to all PLOS ONE policies on sharing data and materials, by including the following statement: "This does not alter our adherence to  PLOS ONE policies on sharing data and materials.” (as detailed online in our guide for authors <http://journals.plos.org/plosone/s/competing-interests>).  If there are restrictions on sharing of data and/or materials, please state these. Please note that we cannot proceed with consideration of your article until this information has been declared.

Response: We have added that statement to the competing interest section of the cover letter and have added a statement on requesting the data.

Please include your updated Competing Interests statement in your cover letter; we will change the online submission form on your behalf.

Response: We have done so.

3. In your Data Availability statement, you have not specified where the minimal data set underlying the results described in your manuscript can be found. PLOS defines a study's minimal data set as the underlying data used to reach the conclusions drawn in the manuscript and any additional data required to replicate the reported study findings in their entirety. All PLOS journals require that the minimal data set be made fully available. For more information about our data policy, please see <http://journals.plos.org/plosone/s/data-availability>.

"Upon re-submitting your revised manuscript, please upload your study’s minimal underlying data set as either Supporting Information files or to a stable, public repository and include the relevant URLs, DOIs, or accession numbers within your revised cover letter. For a list of acceptable repositories, please see <http://journals.plos.org/plosone/s/data-availability#loc-recommended-repositories>. Any potentially identifying patient information must be fully anonymized.

Important: If there are ethical or legal restrictions to sharing your data publicly, please explain these restrictions in detail. Please see our guidelines for more information on what we consider unacceptable restrictions to publicly sharing data: <http://journals.plos.org/plosone/s/data-availability#loc-unacceptable-data-access-restrictions>. Note that it is not acceptable for the authors to be the sole named individuals responsible for ensuring data access.

We will update your Data Availability statement to reflect the information you provide in your cover letter.

Response: We obtained the data, with permission, from the Michigan Genomics Initiative. We have contact information for the Michigan Genomics Initiative so that others may similarly apply for the data. **Data Availability:** Data are available from the Michigan Genomics Initiative https://precisionhealth.umich.edu/our-research/michigangenomics/#request.

[Note: HTML markup is below. Please do not edit.]

Reviewers' comments:

Reviewer's Responses to Questions

**Comments to the Author**

1. Is the manuscript technically sound, and do the data support the conclusions?

The manuscript must describe a technically sound piece of scientific research with data that supports the conclusions. Experiments must have been conducted rigorously, with appropriate controls, replication, and sample sizes. The conclusions must be drawn appropriately based on the data presented.

Reviewer #1: Yes

Reviewer #2: Yes

2. Has the statistical analysis been performed appropriately and rigorously?

Reviewer #1: Yes

Reviewer #2: Yes

3. Have the authors made all data underlying the findings in their manuscript fully available?

The [PLOS Data policy](http://www.plosone.org/static/policies.action#sharing) requires authors to make all data underlying the findings described in their manuscript fully available without restriction, with rare exception (please refer to the Data Availability Statement in the manuscript PDF file). The data should be provided as part of the manuscript or its supporting information, or deposited to a public repository. For example, in addition to summary statistics, the data points behind means, medians and variance measures should be available. If there are restrictions on publicly sharing data—e.g. participant privacy or use of data from a third party—those must be specified.

Reviewer #1: No

Reviewer #2: Yes

Response: We have added this **Data Availability:** Data are available from the Michigan Genomics Initiative https://precisionhealth.umich.edu/our-research/michigangenomics/#request.

4. Is the manuscript presented in an intelligible fashion and written in standard English?

PLOS ONE does not copyedit accepted manuscripts, so the language in submitted articles must be clear, correct, and unambiguous. Any typographical or grammatical errors should be corrected at revision, so please note any specific errors here.

Reviewer #1: Yes

Reviewer #2: Yes

5. Review Comments to the Author

Please use the space provided to explain your answers to the questions above. You may also include additional comments for the author, including concerns about dual publication, research ethics, or publication ethics. (Please upload your review as an attachment if it exceeds 20,000 characters)

Reviewer #1: Engoren et al study here the genetic variants associated with two international consensus definitions of sepsis (Sepsis 2 and Sepsis 3).To that end they analyze by GAWS multiple genetic variants associated with sepsis from stored DNA of > 18 years old adult patients undergoing surgery at Michigan University Medical Center. Electronic medical records were searched for sepsis using the sepsis-2 and sepsis-3 definitions. In addition microbiological cultures, antibiotics use and ≥2 SIRS or SOFA points were also recorded. Controls did not fulfill the International Classification of Diseases Code for sepsis, SIRS or infection. The authors found 25 genetic variants present in both the sepsis-2 and sepsis-3 groups. They consider that most genetic variants have small effect sizes but cumulatively the polygenic risk scores have good discrimination.
The manuscript is enticing and the underlying idea of considering sepsis as a polygenic disease like hypertension is very attractive, correct in my opinion and worth exploring further.
Major points
1.My main concern regards the selection of the controls as individuals ≥18 years that did not develop sepsis. I understand as stated in Material and Methods (Page 6, lines 125) that patients and controls were followed from January 28, 2008 through June 26, 2016. Is that correct? Eight years of follow-up is a period long-enough to detect sepsis predisposition in old individuals (for instance ≥75 years) but too short for 30 years old individuals that underwent a surgery (perhaps a minor orthopedic surgery) at Michigan University Medical Center. Younger controls might develop sepsis many years after finishing the 8 years follow-up period. Could you clarify this point?

Response: We agree that controls can later develop sepsis. We have expanded our Limitations to discuss this. It now reads “As we don’t have access to medical records for treatment received at other hospitals, we may have missed episodes of sepsis or infection treated elsewhere. Similarly, control patients may develop sepsis after the analysis. This may bias our results in unknown ways.”

2.Could you compare sepsis and control individuals regarding age, sex and main comorbidities linked to sepsis (diabetes, cancer, HIV, other immunodepressions) in one initial table?. This way the reader could see that both arms, septic and control , are well balanced.

Response: Unfortunately, the data we received are deidentified and without age, sex, and comorbidities and we are unable to obtain that data.

3.Could you analyze the effect of these genetic variants studied on sepsis mortality as others did ?(References 14 and 42)

Response: We have added this as Supplemental Table 3. We describe this in the Methods, Results, and Discussion sections.

Reviewer #2: The manuscript is about an interesting and significant topic, the genetic predisposition to sepsis. Presented original research is within scope of the journal. Clear, professional English language is used in the text.
There is appropriate Introduction to show context and own results. Literature is well referenced and relevant. Structure of this manuscript is in line with the PLOS ONE standards; supplementary material containing tables and figures is relevant, high quality, well labeled and described.
Research question is well and precisely defined. The primary purpose of this study was to create a polygenic risk score for sepsis and to assess its discrimination; the secondary purpose it to identify those genetic variants most associated with the polygenic risk score.
Investigation has been performed in agreement to high technical and ethical standards, and methods are described well, with sufficient information to replicate.
In this GWAS study authors analyzed about 14 million SNPs in a group of 2261 patients with sepsis and more than 13.000 controls founding 772 genetic variants associated with Sepsis-2 and 442 with Sepsis-3 criteria. After multivariate adjustment, 100 variants on 85 genes were associated with Sepsis-2 and 69 variants in 54 genes with Sepsis-3; twenty-five variants were present in both sepsis groups. Most variants showed small effect sizes but cumulatively, the polygenic risk scores had good discrimination.
The results of this study could have practical implementation; based on obtained results, polygenic risk scores could be further explored for diagnostic and prognostic purposes. This is important because sepsis is frequent cause of mortality and morbidity with still unexplained determinants.
The authors critically discussed some limitations of the study linked mainly to the selection criteria for studied population (ethnical origin of patients with sepsis, medical history of both patients and controls). The specificity of this study is that the sepsis patients were obtained from careful review of the medical record to find patients who met either Sepsis-2 or Sepsis-3 criteria. That is important because studies of sepsis that use administrative records, such as ICD codes, miss many cases of sepsis, leading to the biases in GWAS.
All underlying data have been provided; they are sufficient, statistically correct and controlled. Contemporary bioinformatics tools have been used adequately and expertly.
Conclusions are well stated, linked to original research question and to supporting results. The main conclusion is that that established polygenic risk scores predict sepsis with good discrimination.
Authors found 95 variants on 72 genes to be independently associated with Sepsis-2 and 68 variants in 54 genes to be associated with Sepsis-3 criteria.

I suggest accepting this manuscript in present form; no modifications are required.

Response: Thank you for your kind comments.

6. PLOS authors have the option to publish the peer review history of their article ([what does this mean?](https://journals.plos.org/plosone/s/editorial-and-peer-review-process#loc-peer-review-history)). If published, this will include your full peer review and any attached files.


If you choose “no”, your identity will remain anonymous but your review may still be made public.


**Do you want your identity to be public for this peer review?** For information about this choice, including consent withdrawal, please see our [Privacy Policy](https://www.plos.org/privacy-policy).

Reviewer #1: No

Reviewer #2: **Yes:** Full Professor Maja Surbatovic, MD, PhD

[NOTE: If reviewer comments were submitted as an attachment file, they will be attached to this email and accessible via the submission site. Please log into your account, locate the manuscript record, and check for the action link "View Attachments". If this link does not appear, there are no attachment files.]

While revising your submission, please upload your figure files to the Preflight Analysis and Conversion Engine (PACE) digital diagnostic tool, <https://pacev2.apexcovantage.com/>. PACE helps ensure that figures meet PLOS requirements. To use PACE, you must first register as a user. Registration is free. Then, login and navigate to the UPLOAD tab, where you will find detailed instructions on how to use the tool. If you encounter any issues or have any questions when using PACE, please email PLOS at [figures@plos.org](mailto:figures@plos.org). Please note that Supporting Information files do not need this step.
